# Clinical Use of S53P4 Bioactive Glass in the Treatment of Bone Defects and Infected Bone: A Systematic Review of the Quality of Clinical Outcomes and A Grade Assessment

**DOI:** 10.1002/adhm.202503013

**Published:** 2025-12-03

**Authors:** Sebastian CE Lindfors, Chris JJ Arts, Nina C Lindfors

**Affiliations:** ^1^ Helsinki University Helsinki University Hospital Department of Musculoskeletal and Plastic Surgery Helsinki Finland; ^2^ Department of Orthopedic Surgery Maastricht University Medical Centre the Netherlands; ^3^ Orthopedic Biomechanics Department Biomedical Engineering Eindhoven University of Technology the Netherlands

**Keywords:** bioactive glass, bone graft substitute, bone regeneration, clinical evidence, infection treatment, osteomyelitis, S53P4

## Abstract

Bioactive glass (BAG) S53P4 is a synthetic bone substitute consisting of oxides of silicon, sodium, calcium and phosphorus and exhibits both osteoconductive and antibacterial properties. Clinically it has been used in the treatment of benign bone tumor surgery, in spine surgery, in trauma surgery, in frontal sinus surgery, in diabetic foot osteomyelitis surgery, in mastoid surgery, in oral and maxillofacial surgery, and in the treatment of bone infections with excellent clinical outcomes. A systematic review following PRISMA guidelines is performed to evaluate the level of evidence, clinical efficacy, and safety of BAG S53P4 as a bone graft substitute in the treatment of bone defects and infected bone. Clinical studies published between 1990 until 2024 are extracted and analyzed (*N* = 99). The highest level of clinical evidence (L1‐L2) of successful use of BAG S53P4 is found in the treatment of benign bone tumor surgery (*N* = 5), in oral and maxillofacial surgery (*N* = 5), in the treatment of bone infection (*N* = 6), in spinal surgery (*N* = 3), and in trauma surgery (*N* = 3). The highest evidence on successful treatment in respect to the number of publications on BAG S53P4 are found in the categories of infection treatment (*N* = 24) and in mastoid surgery (*N* = 30). Derived from this systematic literature review and level of evidence assessment, BAG S53P4 is a safe and effective alternative to autograft bone and offers excellent long‐term outcomes in various clinical indications when bone grafts are needed. In the management of osteomyelitis in infected non‐unions, mastoid surgery and diabetic foot, BAG S53P4 demonstrates high infection eradication rates and successful bone healing. Considering the increasing incidence of microbial resistance to antibiotics its role may become critical in the fight against antimicrobial resistance.

## Graphical abstract

Bioactive glass (BAG) S53P4 is a synthetic bone substitute. Clinically it has been used in the treatment of benign bone tumor surgery, in spine surgery, in trauma surgery, in frontal sinus surgery, in diabetic foot osteomyelitis surgery, in mastoid surgery, in oral and maxillofacial surgery in more than 4000 patients, with excellent clinical long‐term results.

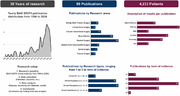

## 1 Introduction

Bioactive glass (BAG) S53P4 is a synthetic bone graft substitute that has gained clinical interest due to its unique combination of osteoconductive and antibacterial properties.^[^ ^1^ ^]^ Composed of silica (SiO_2_), sodium oxide (Na_2_O), calcium oxide (CaO), and phosphorus pentoxide (P_2_O_5_), BAG S53P4 interacts with physiological fluids to form a silica layer and on top a hydroxyapatite layer, which is chemically similar to the mineral phase in bone. The ionic surface reactions at the glass surface with the subsequent increase in pH resulting from ion dissolution of the BAG, also create an environment unfavorable for bacterial growth demonstrated in vitro for a variety of both gram positive and Gram‐negative pathogens.^[^ ^2^, ^3^ ^]^

Initially developed to promote bone growth and bone regeneration, BAG S53P4 is today utilized across a diverse array of surgical disciplines to address bone defects, manage complex fractures, combat infections, and facilitate reconstruction. It serves to fill bone voids, provide structural support, and enhance bone healing, often reducing or eliminating the need for autologous bone (AB) graft harvesting and its associated donor site morbidity.

BAG S53P4 has emerged as a reliable bone graft substitute in the management of benign bone tumors showing comparably results to AB and allograft bone in terms of long‐term bone healing in adults.^[^ ^4^, ^5^, ^6^ ^]^ Promising results regarding safety, bone remodeling, and bone growth have also been reported in the treatment of bone tumors in adults and in pediatric bone tumor surgery.^[^ ^7^, ^8^, ^9^ ^]^

BAG S53P4 is a well‐tolerated material, in oral and maxillofacial surgery in reconstruction of bone defects following tumor resection and in orbital floor repair.^[^ ^10^, ^11^ ^]^ In spinal surgery, BAG S53P4 has been utilized as a bone graft extender showing good clinical and radiological results in the terms of improved clinical outcomes and fusion rates in both degenerative conditions and traumatic injuries.^[^ ^12^, ^13^ ^]^ In trauma surgery, BAG S53P4 has also demonstrated significant clinical utility in managing intra‐articular fractures, particularly those complicated by infection, bone loss, or non‐union. It serves to fill bone voids, support structural integrity, and promote bone regeneration.^[^ ^14^, ^15^ ^]^

The use of BAG S53P4 has been highly effective as part of the treatment of infected bone. Initiated by frontal sinus surgery, BAG S53P4 has proven to be a biocompatible and useful bone graft material, with a high success rate in achieving an uneventful recovery.^[^ ^16^ ^]^ Comparable favorable results with low infection recurrence rates have also been noted in the treatment of mastoiditis.^[^ ^17^ ^]^

Osteomyelitis is a challenging complication in orthopedic and trauma surgery. The osteoconductive and antimicrobial properties of BAG S53P4 makes it particularly valuable in the treatment of bone infection. Since its initial reported use in osteomyelitis in 2010, while showing a successful outcome in 10/11 patients,^[^ ^18^ ^]^ numerous studies have reported high rates of infection eradication up to 90%^[^ ^19^ ^]^ as well as successful bone integration in the treatment of infected non‐unions.^[^ ^20^ ^]^ In the specific context of diabetic foot osteomyelitis,^[^ ^21^ ^]^ BAG S53P4 has shown favorable results. Studies report successful integration, infection resolution, and improved healing in patients with complex ulcers and Charcot neuroarthropathy.^[^ ^22^ ^]^

The growing body of clinical evidence underscores BAG S53P4 as a versatile and highly effective synthetic bone graft substitute. Its consistent success in promoting bone regeneration while concurrently providing an antibacterial effect makes it valuable in managing a wide range of challenging clinical situations, from bone tumor defects and spinal fusions to complex trauma, chronic infections like osteomyelitis, and specialized reconstructions in various surgical fields. By minimizing the need for AB and supporting long‐term healing and infection control, BAG S53P4 continues to solidify its role as a valuable biomaterial in modern regenerative and reconstructive surgery.

This systematic review examines published BAG S53P4 data from 1990 to 2024 and evaluates the level of evidence, clinical efficacy, and safety of BAG S53P4 as a bone graft substitute in the treatment of bone defects and infected bone.

## 2 Experimental Section

**FIGURE 1:**
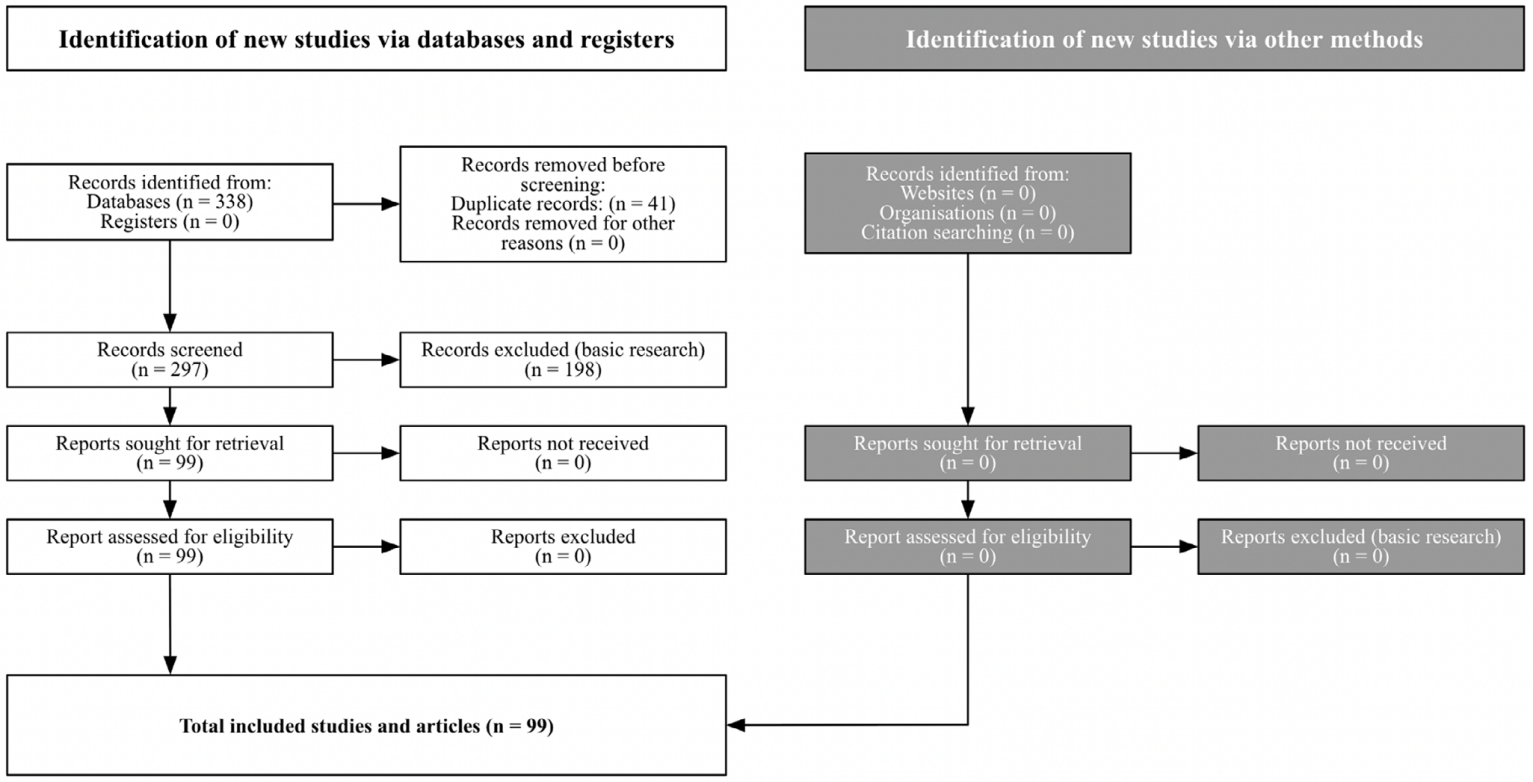
Flow diagram showing identification of publications via databases and registers according to the PRISMA 2020 flow diagram for new systematic reviews, including databases, registers, and other sources (PRISMA 2020 flow diagram—PRISMA statement).

Clinical data on the specific BAG S53P4 (Bonalive) from 1990–2024 were systematically identified by searches in databases PubMed and Scopus, registers and organizational publications such as doctoral theses (**Figure** **1**) using the keywords: bioactive glass and S53P4 and bone substitute. Afterwards the studies were categorized into Level of evidence by the reviewers (SL, NCL) and in case of disagreement a third reviewer was consulted (CA). The Oxford OCEBM classification of levels of evidence was used, L1: high‐quality randomized trials or prospective studies, L2: lesser methodological quality prospective studies of Level 2 studies, L3: case control and retrospective comparative studies, L4: case series, and L5: single patient study.^[^ ^23^ ^]^

## 3 Results

In total 99/338 publications including benign bone tumor surgery, frontal sinus surgery, oral and maxillofacial surgery, bone infection, mastoid surgery, diabetic foot osteomyelitis surgery, spinal surgery and trauma surgery were accepted in the study according to the flow diagram (Figure 1). The non accepted publications fell in the category of basic science representing in vitro, in vivo studies and mechanical studies.

**FIGURE 2:**
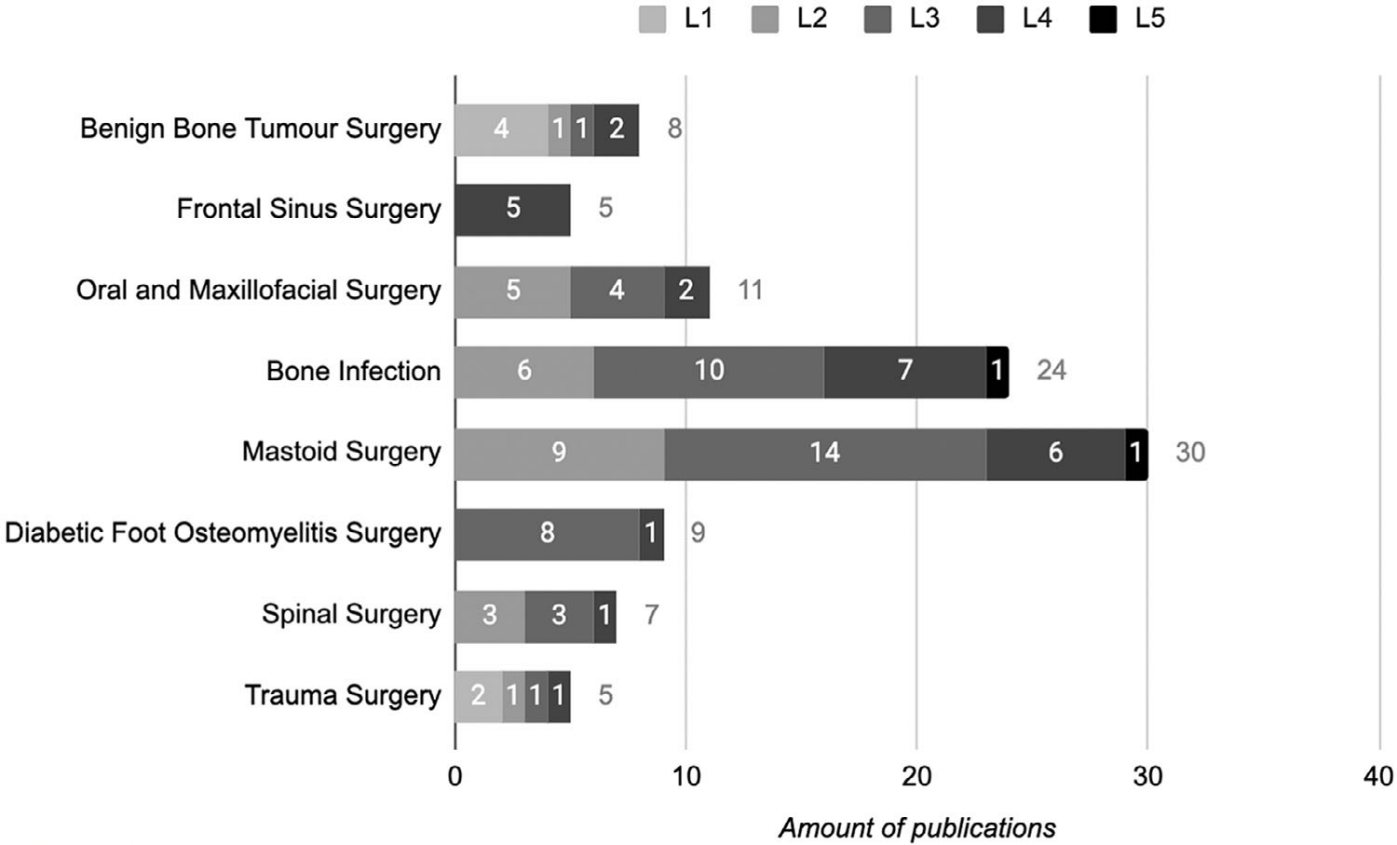
Medical indication of using BAG S53P4 in relation to level of evidence (L1‐L5) (*N* = 99).^[^ ^23^ ^]^

**FIGURE 3:**
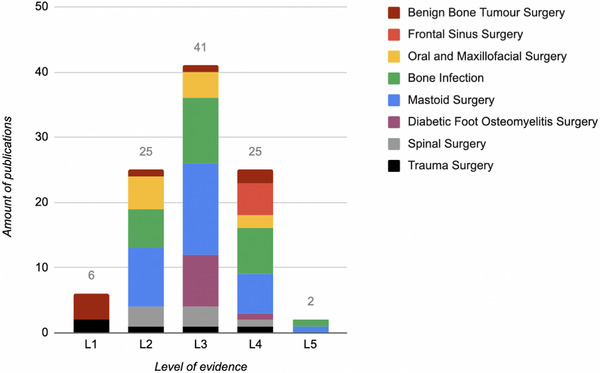
Level of evidence (L1‐L5) on using BAG S53P4 in relation to medical indication (*N* = 99).^[^ ^23^ ^]^

**FIGURE 4:**
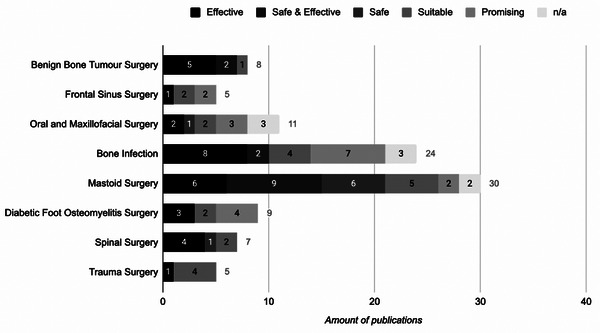
The clinical outcome on using BAG S53P4 described in the publications for various medical indications (*N* = 99).

The highest level of evidence (L1‐L2) of using BAG S53P4 in the treatment of bone defects, is seen in benign bone tumor surgery (*N* = 5), oral and maxillofacial surgery (*N* = 5), in the treatment of bone infection (*N* = 6), in spinal surgery (*N* = 3), and in trauma surgery (*N* = 3) (**Figure** **2**). In respect to the number of publications strengthening the evidence of the use of BAG S53P4, most publications are found in the categories of infection treatment (*N* = 24) and in mastoid surgery (*N* = 30), making infection treatment the most important treatment indication for using BAG S53P4. This is also in concordance with the number of publications and different levels of evidence (L2‐L4), when also including mastoid surgery, diabetic foot osteomyelitis surgery, oral and maxillofacial surgery, and frontal sinus surgery (**Figure** **3**). The clinical outcome of the treatment using S53P4 described by the authors in a publication i.e. effective, safe and effective, safe, suitable, and promising in each publication is summarized in **Figure** **4**.

## 4 Discussion

The search for effective bone graft substitutes has driven significant advancements in biomaterials in bone reconstructive surgery. Among these promising bone substitutes, BAG S53P4 originally developed to address the limitations of traditional autografts and allografts—such as donor site morbidity and limited supply, has emerged as a versatile and clinically valuable alternative. Its unique bioactive properties stimulate bone regeneration making it suitable for a wide range of surgical indications from benign bone lesions to complex infections and craniofacial reconstructions.

### 4.1 Bone Tumor Surgery

Bioactive glass S53P4 has shown to be a safe, effective and long‐lasting alternative to autograft or allograft bone grafts supporting bone regeneration and healing in the treatment of benign bone lesions including enchondromas, aneurysmal bone cysts, and other benign tumors in both adults and children. Several prospective randomized trials and clinical trials show similar good overall treatment success rates while comparing autograft and allografts with BAG S53P4.

In a prospective randomized controlled trial using AB and BAG S53P4 for the treatment of benign tumors, the filled cavity was replaced faster by new bone in the AB group than in the BAG group (*p* = 0.0001), however at 3 years, no statistical difference in cavity volume between the two groups was observed on X‐rays (*p* = 0.7881) or on CT scans (*p* = 0.9117). In the BAG group, the filled cavity appeared dense on X‐rays, and glass granules were still observed on CT. The cortical thickness seemed to have increased more in the BAG group than in the AB group (*p* < 0.0001).^[^ ^4^ ^]^ Comparing clinical efficacy of BAG S53P4 granules and stand alone bone grafts (autograft for small‐sized defects and allografts for large defects) in 49 randomized patients, 21 of 25 BAG‐treated patients and 20 of 24 patients in the standard of care group met the criteria for treatment success including similar Rand‐36 scores. In patients with small defects, BAG S53P4 filling was associated with shorter operative time and less postoperative pain at one month. In patients with large defects BAG S53P4 filled defects showed higher metabolic activity compared to allograft‐filled defects at one year, confirming cellular responses of osseointegration of BAG S53P4 granules.^[^ ^5^ ^]^

These promising results are confirmed in a prospective randomized 14‐year long‐term follow‐up study comparing BAG S53P4 and AB in the treatment of benign bone tumors, showing a mainly or partly fatty bone marrow on MRI in the large bone tumor group and an increased cortical thickness in non‐ossifying fibromas and enchondromas confirming that BAG S53P4 is a safe and well‐tolerated bone substitute with good long‐term results.^[^ ^6^ ^]^ Comparable results have been reported in the treatment of bone tumors in the lower extremities, showing that BAG S53P4 is an excellent bone substitute that can be used as a replacement of autografts with minimal access and exposure of the bone thus expected a faster recovery as well as minimizing the risk of infections and excessive bleeding.^[^ ^24^ ^]^ In a retrospective comparative study on surgically treated enchondromas in the hand during a 17‐year period on 190 patients (116 AB and 74 BAG) including 205 enchondromas, no statistically significant differences in outcome measures were observed. A reoperation was performed in five patients in the AB group and none in the BAG group, suggesting that BAG S53P4 is a safe and effective bone‐graft material alternative for filling of enchondroma‐evacuated cavities.^[^ ^25^ ^]^

The first report on using BAG S53P4 in children was on the treatment of a recurrent aneurysmal bone cyst showing good bone remodeling and normal growth of the phalange during a two‐year follow‐up.^[^ ^7^ ^]^ Similar results have been reported on 18 consecutively treated children with histologically proven primary aneurysmal bone cysts treated with BAG S53P4 (mean 11.3 years at surgery, 11 lower limb, 6 upper limb, 1 pelvis, and 15 with primary surgery). Bone remodeling without growth plate disturbance was observed in all patients. A recurrence was observed in two children.^[^ ^8^ ^]^

In a randomized study morselized allograft (*N* = 26) and BAG S53P4 (*N* = 25) was compared in the treatment of pediatric benign bone lesions including simple bone cysts (*N* = 14), aneurysmal bone cysts (*N* = 10), a nonossifying fibroma (*N* = 1) and a fibrous dysplasia (*N* = 1) in children (*N* = 64, 4–16 years, mean 11.1 years) with comparable lesion sizes in both groups. In the allograft group (*N* = 12) and in the BAG group (*N* = 10) developed a recurrence. The outcome of the study was that filling benign bone lesions in children with either allograft or BAG S53P4 provides comparable results in terms of recurrence and complications. BAG S53P4 was regarded as a safe and efficient alternative to allograft for the treatment of pediatric bone lesions.^[^ ^9^ ^]^

For bone tumors the overall evidence demonstrates that BAG S53P4 is a highly viable alternative to autografts and allografts in the treatment of benign bone lesions. It offers several advantages, including biocompatibility, structural stability, reduced surgical morbidity, and favorable long‐term outcomes, with comparable or superior performance across a range of lesion types, defect sizes, and patient populations, including pediatric bone reconstruction.^[^ ^8^ ^]^

### 4.2 Spinal Surgery

BAG S53P4 has increasingly gained attention as a viable bone graft material in spinal surgery, both as a stand‐alone substitute and as a graft extender when combined with autogenous bone (AB). Clinical studies have evaluated its performance across a variety of spinal conditions, surgical techniques, and patient populations, demonstrating promising fusion rates, safety, and good clinical outcomes.

In a prospective 11‐year long‐term follow‐up study on BAG S53P4 and AB used as bone graft material implanted on either side of the spine for the treatment of degenerative spondylolisthesis, a solid bony fusion was seen on CT scans in the AB group in all patients and in the BAG group in 12/17 patients. The fusion rate of all fusion sites (*n* = 41) for BAG S53P4 was 88% at L4/5 and 88% at L5/S1. The mean Oswestry Disability Index score was 21 (0–52), compared to 49 (32–64) at the preoperative time.^[^ ^12^ ^]^ In a comparable 10‐year prospective randomized follow‐up study comparing BAG S53P4 and AB in the treatment of lumbar burst fractures, a solid bony fusion was observed on CT scans in the AB side in 10/10 patients and on BAG side in 5/10 patients and a partial fusion in 5/10 patients, resulting in a total fusion‐rate of 71% of all fused segments in the BAG group. The Oswestry score was excellent and the mean pain score was 1.^[^ ^13^ ^]^ Comparable results have been reported in a retrospective analysis on 20 patients, who had undergone a minimally invasive transforaminal lumbar interbody fusion using BAG S53P4 putty as a bone graft extender together with local AB, an interbody fusion rate of 95.8% was achieved.^[^ ^26^ ^]^ In a retrospective case series study on 39 patients who underwent primary multilevel instrumented two‐level (43%), three‐level (31%), four‐level (23%), and five‐level (3%) fusion surgery using BAG S53P4 in combination with a porous PEEK interbody spacer for the treatment of degenerative cervical disc disease, reporting that a cervical fusion with an improved neurological status was observed for all patients.^[^ ^27^ ^]^ Evaluating the postoperative safety and efficiency of using stand‐alone BAG S53P4 putty and granules in posterior spinal fusion in the treatment of scoliosis in children and adolescents (*N* = 43), no significant loss of correction between the immediate postoperative time point could neither be observed during a 24‐month follow‐up, nor any sign of non‐union, implant displacement or rod breakage.^[^ ^28^ ^]^

In the treatment of infected bone a posterolateral spondylodesis, using transpedicular fixation, performed posteriorly combined with an anterior decompression and reconstruction using an expandable replacement device and BAG S53P4 (*N* = 2) and BAG S53P4 mixed with autograft bone (*N* = 1), were performed due to spondylodiscitis caused by *Mycobacterium tuberculosis, Candida tropicalis*, or *Staphylococcus aureus*. Complete fusion and neurological recovery was observed in all patients.^[^ ^29^ ^]^ Comparable good results have also been achieved using BAG S53P4 as a posterolateral graft material in patients with infected spondylodesis.^[^ ^30^ ^]^

### 4.3 Trauma Surgery

The application of BAG S53P4 in trauma surgery has shown good results both in acute fracture management and in the treatment of complex bone healing disorders such as non‐unions. In a prospective randomized 11‐year study on depressed tibial plateau fractures, the mean articular surface depression on X‐rays were 1.4 (0–2) mm in the BAG group and 1.4 (0–4) mm in the AB group, and on CT scans 2.2 (2–3) mm, and 2.1 (0–3) mm respectively. No significant difference in the tibial‐femoral angle or the deviation of the mechanical axes was observed between the two groups, revealing that BAG‐S53P4 can be used as a bone substitute in depressed lateral tibial plateau fractures with good functional and radiological long‐term results.^[^ ^14^ ^]^ Comparable good results have been reported at 1 year, showing no differences in subjective evaluation, functional tests or clinical examination at a 1‐ year follow‐up.^[^ ^31^ ^]^

Managing bone non‐unions in long bones remains challenging, especially in defects exceeding 5 cm size. BAG S53P4 has gained increasing attention in managing complex fractures as well as in the treatment of non‐unions. A combination of BAG S53P4 and autologous bone (RIA) has shown to be a usable filler material for the treatment of non‐unions in both femur and tibia, showing complete consolidation at one year in *N* = 6/13 patients (46.2%). Three patients (23.1%) had evident callus formation with expected stability according to the Lane‐Sandhu score.^[^ ^32^ ^]^ A similar promising good result has also been reported for a supracondylar femoral non‐union using morselized bone and BAG S53P4.^[^ ^33^ ^]^ In the treatment of infected tibial nonunion equally good results have been reported using the Illizarov technique and BAG S53P4 during a follow‐up of 113 weeks. According to the ASAMI Functional Scoring system 38.5% of the results were graded excellent and 46.1% as good, and according to the Radiological Scoring Systems the outcome was excellent in 61.5% and good in 34.6% of the patients.^[^ ^15^ ^]^

### 4.4 Frontal Sinus Surgery

BAG S53P4 has shown to be a reliable and well tolerated obliteration material in frontal sinus surgery both in short and long‐term follow‐up.^[^ ^34^, ^35^, ^36^ ^]^

In a series of osteoplastic frontal sinus operations on patients suffering from chronic frontal sinusitis, which had not been cured with other means of treatment, accurate obliteration of sinuses was achieved in 42/39 patients and an uneventful recovery and clinical outcome in 92% of the patients, without growth of bacteria. Fourier‐transform infrared (FTIR) studies showed bone produced around BAG S53P4 to be similar to natural frontal bone.^[^ ^37^ ^]^ In a comparable series of osteoplastic frontal sinus operations on 32 patients from 1990 to 1999, five consecutive patients were controlled by CT scans at one week, 6, 12, 24, 36, 48 months. Eight years after obliteration the clinical status and CT scans were normal and the histopathologic examination showed a clear woven bone formation without any inflammatory or foreign body reactions.^[^ ^15^ ^]^ Similar favorable results have been reported for BAG S53P4 with complete obliteration of all sinus recesses and excavations, without adverse effects of the implant material over a mean follow‐up period of 5.0 years.^[^ ^37^ ^]^

### 4.5 Diabetic Foot

Osteomyelitis in diabetic patients is difficult to treat and often leads to amputation of limbs, and due to the increasing prevalence of diabetes, it has become a global health problem. BAG S53P4 has also found its way as part of the treatment of infected bone in diabetic patients. It has been successfully used in the treatment of osteomyelitis of the cubital bone due to chronic foot ulcer, in a chronic ulcer apical‐plantar of the left big toe with low tendency to healing and in the limb‐salvage staged treatment of a patient with an infected Charcot foot, showing full integration of BAG S53P4 with the surrounding bone.^[^ ^22^, ^38^, ^39^ ^]^

Clinical reports and case series have demonstrated that BAG S53P4 can be effectively used to manage infected bone in diabetic foot complications, including osteomyelitis and septic osteoarthritis.^[^ ^40^ ^]^ Individual case reports have highlighted successful integration of BAG S53P4 into the surrounding bone, with radiological and clinical evidence of infection resolution and improved wound healing even in anatomically and pathologically complex scenarios such as Charcot neuroarthropathy and chronic nonhealing ulcers.

In a retrospective case series including 16 patients with diabetic foot complications and septic osteoarthritis, BAG 53P4 was used as part of the treatment, in which eight patients received treatment of the metatarsophalangeal joints, four patients of the metatarsal joints and four patients of the ankle. Ten patients suffered from Charcot neuroarthropathy. None of the patients required readmission within 30 days or suffered from early complications. Similar successful results on patients (*N* = 10) treated for complicating infection of diabetic foot, showing a healing rate of 80% in 34 days, with only one patient needing a second look.^[^ ^41^ ^]^ Equally good results have been reported in a retrospective observational study on 22 patients with septic osteoarthritis. Ten patients (group A) were treated with segmental resection of the first MTP joint and periarticular bone, stabilization with an external fixator, and a local application of BAG S53P4 mixed with 5 mL of venous blood and 12 patients (group B) were treated with combined temporary application of a Septopal Chain for 3 weeks. Successful healing with a complete resolution of osteomyelitis was achieved in all 10 patients in group A and in 9/12 patients in group B. The addition of BAG S53P4 seemed to enable greater stability and with fewer late complications, e.g. hallux valgus.^[^ ^42^ ^]^ Similar excellent primary outcomes of using BAG S53P4 has been associated with an 81% lower probability of needing additional antibiotic therapy compared to subjects treated with a traditional procedure,^[^ ^21^ ^]^ no sign of persistent infection and a healing rate of 66.6% due to skin and vascular complications.^[^ ^43^ ^]^ In a rare case of the syndrome of Guillain–Barrè a 19‐year‐old girl suffering from a chronic plantar hindfoot‐infected ulceration since 2 years of age was treated with BAG S53P4 with successful outcome, showing complete wound healing.^[^ ^44^ ^]^

### 4.6 Bone Infection

Bone infection e.g. osteomyelitis, represents a persistent challenge in orthopedic and trauma surgery due to its chronic nature and the resistance to standard antimicrobial treatments. Traditional management strategies typically involve a combination of systemic antibiotic therapy, surgical debridement, followed by reconstruction of the bone tissue using autografts, allografts or bone substitutes.

Clinical studies have demonstrated the efficacy of BAG S53P4 in managing chronic osteomyelitis, infected non‐unions, and posttraumatic bone infections. Its use has been associated with high rates of infection eradication, biocompatibility, and favorable integration with the surrounding bone tissue shown in case reports and small cases series: a rare fungal osteomyelitis case in the calcaneus caused by paracoccidioidomycosis,^[^ ^45^ ^]^ Brodie's abscess,^[^ ^46^ ^]^ and an infected nonunion of the humerus,^[^ ^47^ ^]^ as well as in retrospective and prospective clinical trials including both children and adults.^[^ ^48^, ^49^, ^50^, ^51^ ^]^

BAG S53P4 used in the treatment of osteomyelitis was first published in 2010 with excellent results.^[^ ^18^ ^]^ This study was followed by a multicenter study on 116 patients with a 90% success rate.^[^ ^19^ ^]^ The observed favorable clinical outcome has opened new treatment methods and an increasing interest in using BAG S53P4 in the field of bone infection. In a retrospective, observational study on patients (*N* = 31) with a clinical and radiological diagnosis of chronic osteomyelitis (47.1%, *Staphylococcus aureus*), treated with BAG S53P4, 90.3% of the patients were classified as “disease‐free” after a one‐year follow‐up, showing that the BAG S53P4 is a safe and effective bone substitute to treat cavitary chronic osteomyelitis, including infections caused by resistant pathogens, such as methicillin‐resistant *S. aureus*.^[^ ^52^ ^]^ Comparable results have been reported in a multicenter observational study on patients undergoing surgical treatment for chronic cavitary osteomyelitis, showing an infection eradication of 85.9% and 87.2% at 6‐month and 12‐month follow‐ups, respectively.^[^ ^53^ ^]^ Equally good results have been reported in a prospective multicenter study on 78 patients with chronic cavitary long bone osteomyelitis of which 69 patients were treated in a one‐stage procedure the overall infection eradication was 85% and 1‐year infection‐free survival 89%.^[^ ^51^ ^]^ The results are also in concordance with a study on 27 patients with a clinically and radiologically diagnosed osteomyelitis of long bones, showing no sign of infection in 88.9% of the patients.^[^ ^54^ ^]^ Equal healing rate of 92% has reported for 24 patients with Cierny–Mader type 3 osteomyelitis treated with BAG S53P4,^[^ ^55^ ^]^ as well as for the management of chronic osteomyelitis of the appendicular skeleton showing an overall resolution rate of 94% (*N* = 80) on using single‐stage surgeries and tailored dead space management strategies in South Africa.^[^ ^56^ ^]^ The treatment of osteomyelitis is challenging depending not only on the bone graft but the whole surgical procedure as well as radiological evaluation. Lesser success rates of using BAG S53P4 have been reported on the treatment of chronic osteomyelitis in low‐ and middle‐income countries as contemporary treatment options cannot be adapted with the same results in these settings.^[^ ^57^ ^]^

Today BAG S53P4 has not only been used in the treatment of osteomyelitis, but it has also been increasingly utilized as a bone graft substitute in managing infected and septic non‐unions. In a case series on 18 patients with septic non‐unions, six underwent internal osteosynthesis and 12 were treated with external fixators in combination with bioactive glass grafting. Fracture union was achieved according to the RUST score in 17 of 18 cases (94.4%, one patient was lost to follow‐up).^[^ ^58^ ^]^

The preliminary results on using a new treatment algorithm for infected non‐unions of the tibia have also been presented in a retrospective case series. The patients (*N* = 5, mean age 55, average NUSS‐score 44 and the average segmental bone defect 4.6 cm) were treated with extensive debridement surgery, replacement of the osteosynthesis and implantation of S53P4 BAG and BMAC (bone marrow aspirate concentrate) in a one‐stage or two‐stage procedure based on non‐union severity including culture based antibiotic therapy and followed until union and infection eradication. One patient was treated in a one‐stage procedure and four patients in a two‐stage induced membrane‐, or “Masquelet”‐procedure. At the end of the 13.6 month follow‐up all patients had clinical consolidation with an average RUST‐score of 7.8 and complete eradication of infection.^[^ ^59^ ^]^ In another retrospective study using BAG S53P4 in the treatment of 50 patients with osteomyelitis and infected non‐unions, most often caused by *Staphylococcus aureus*, with a follow‐up of 12.3 months, a healed bone defect was observed in 26/37 after 6 months. At 12 months 83.3% of the filled bone defects were healed. Seven patients suffered from a reinfection, no BAG S53P4 associated complications were not seen.^[^ ^20^ ^]^

In a retrospective study comparing BAG S53P4 (*N* = 51) and AB graft (*N* = 32) for filling defects in patients with chronic osteomyelitis and infected non‐unions a reinfection was observed in the BAG group in 15 patients (29%) and in the AB in six patients (19%). Sixty‐four of the patients showed complete bone healing at the end of the follow‐up period (BAG *n* = 39, 77%, AB *n* = 25, 78%). Patients with multidrug‐resistant pathogens had a significantly higher rate of incomplete bone healing (*p* = 0.033) and a 3‐fold higher risk of complications in both groups. The conclusion was that BAG S53P4 appears to be a suitable bone substitute not only for successful control of infection and defect filling but also for bone healing in cases of infected non‐union, being neither superior nor inferior to AG with regard to the primary and secondary endpoints.^[^ ^60^ ^]^ Comparable results have been reported in a retrospective study describing the efficacy of BAG S53P4 in the treatment of chronic osteomyelitis (*N* = 24) and in septic non‐union (*N* = 14). Infection eradication was successful in 22/24 patients (91.7%) in chronic osteomyelitis and in 11/14 patients (78.6%) with septic nonunion achieving both infection and fracture healing in 9.1 ± 4.9 months.^[^ ^61^ ^]^

The favorable results of using BAG S53P4 in the treatment of osteomyelitis have also changed the treatment methods from a two‐staged procedure to a one‐stage procedure.^[^ ^62^ ^]^

In a retrospective study on patients (*N* = 8) from several medical centers in Europe, BAG‐S53P4 was used as part of the treatment in an induced membrane technique to fill the resultant cavity (*N* = 3) as a stand‐alone and (*N* = 5) mixed with autograft, combined with different surgical procedures. At a follow‐up of 16 months, all healed without any recurrence of the infection. The conclusion was that BAG‐S53P4 may be considered as bone graft in an induced membrane technique, especially when there is a high probability of occurrence or recurrence of a bone infection.^[^ ^63^ ^]^

In a retrospective comparative analysis of patients (*N* = 25) with clinically and radiologically diagnosed osteomyelitis, BAG S53P4 was used in 11 patients and calcium sulfate antibiotic beads in 11 patients, an equally effective result was observed for both groups.^[^ ^64^ ^]^ Comparable results have been reported in a retrospective study on the safety and efficacy of surgical debridement and local application of BAG S53P4 (*N* = 27), to antibiotic‐loaded hydroxyapatite and a calcium sulphate compound (*N* = 27) or a mixture of tricalcium phosphate and an antibiotic‐loaded demineralized bone matrix (*N* = 22). The control of infection was similar in all three groups (93%, 89%, 86%), showing that patients treated with BAG S53P4 without local antibiotics achieved similar eradication of infection and less drainage than those treated with two different antibiotic‐loaded calcium‐based bone substitutes.^[^ ^65^ ^]^

### 4.7 Mastoid Surgery

In mastoid surgery, BAG S53P4 has proven to be a well‐tolerated bone substitute showing long‐term stable results, without donor site morbidity. Clinical outcomes show high rates of **s**uccessful cavity obliteration, low incidence of drainage, good outcomes and MRI compatibility, allowing long‐term monitoring of residual disease. A 10‐year single‐center study involving 173 adult patients undergoing cholesteatoma surgery with mastoid obliteration using BAG S53P4, has shown sustained obliteration, good anatomical preservation, and low recurrence rates.^[^ ^17^ ^]^

In a single‐center retrospective cohort study conducted on all (*N* = 97) patients a dry and safe ear was noted in 95% of the patients, thereby enabling the wearing of a conventional hearing aid.^[^ ^66^ ^]^ Comparable results have been received in the treatment of subtotal petrosectomy for chronic suppurative otitis media.^[^ ^67^ ^]^ Obliteration of the mastoid cavity using BAG S53P4 along with mastoidectomy in patients with chronically discharging also significantly improves the achievement of a dry and safe ear as compared to mastoidectomy alone. The use of BAG S53P4 has shown to be a safe well‐tolerated and effective obliteration material in cholesteatoma surgery and an effective technique to avoid cavity problems,^[^ ^68^, ^69^, ^70^, ^71^, ^72^, ^73^, ^74^, ^75^, ^76^, ^77^, ^78^, ^79^ ^]^ as well improving the surgical outcome in noncholesteatoma chronic otitis media patients.^[^ ^80^ ^]^ The favorable outcome of using BAG S53P4 has also been demonstrated in the treatment in a pediatric cholesteatoma cohort, showing a dry ear in almost all patients with minor complications.^[^ ^81^ ^]^ Pediatric Peri‐/postoperative antibiotics have been noted to prevent early infection in obliteration surgery with S53P4 granules.^[^ ^82^ ^]^

Bone dust or granules of BAG S53P4 has proved to be a safe and effective method in the management of chronic suppurative otitis media with cholesteatoma,^[^ ^83^ ^]^ however, a different outcome showing a slightly higher risk on surgical site infection has been reported while combining BAG S53P4 and bone dust.^[^ ^84^ ^]^

Comparing biologic hydroxyapatite and BAG S53P4 in a radiological study have shown to provide a more optimal osseointegration versus BAG S53P4 with no significant differences in graft resorption and clinical tolerance. However, complications namely otological acute or chronic infections, unbalanced metabolic disease, long‐term cortico‐steroid therapy, auto‐immune disease, history of allergy to grafting materials and postsurgery CT scan in other centers (N  =  8) were all excluded from the study.^[^ ^85^ ^]^ High‐resolution computed tomography (HRCT) and MRI to evaluate the appearance of mastoid and epitympanic obliteration attenuation, homogeneity, and osseointegration of BAG S53P4 granules (*N* = 70) have shown a mostly homogenous obliteration with partly osseointegration. Radiological follow‐up of patients operated on with mastoid and epitympanic obliteration with BAG granules has shown to be effective both using HRCT and MRI.^[^ ^86^ ^]^ Comparable results during a follow‐up imaging with 18F‐NaF PET/CT after three years of BAG S53P4 implantation have shown increased uptake in the obliterated cavity, indicating new bone formation.^[^ ^87^ ^]^

These favorable results are strengthened by the results on analyzing anatomical, functional and quality‐of‐life results when using BAG S53P4 for mastoid and epitympanic obliteration in canal‐wall‐down or canal‐wall‐up tympanoplasties, showing that the use of BAG S53P4 for mastoid and epitympanic obliteration is an effective procedure in both primary and revision surgery. The anatomical and functional results also appear correlated well with patient experience and to the improvement in quality of life.^[^ ^88^ ^]^

In a prospective, randomized, single blind comparative study the outcome and efficacy of mastoid obliteration following canal wall down mastoidectomy (*N* = 40), using two different materials, bone pâté and BAG S53P4 was conducted for a period of 2 years in a tertiary care center. A statistically better obliteration was observed in the BAG S53P4 group, showing a better outcome for effectiveness of obliteration compared to bone pâté.^[^ ^89^ ^]^ In a randomized study (*N* = 68) on a single‐stage canal wall down mastoidectomy with mastoid obliteration using either bone pâté (*N* = 35) or BAG S53P4 (*N* = 33), with a follow‐up of 12 months, a dry epithelized cavity was achieved in 96%, showing no significant difference between the two groups, except for a shorter duration of surgery in the BAG group.^[^ ^90^ ^]^

A favorable observation on using BAG S53P4 is also noted among patients who have had mastoid obliteration with BAG S53P4, as the procedure enables safe placement of a Bonebridge implant later in difficult anatomical conditions.^[^ ^91^, ^92^, ^93^ ^]^

In a 30‐year long‐term study analyzing 843 cases of mastoid obliteration from the pool of data on 16 000 surgical procedures on the ear, including materials of cartilage/bone, Palva flaps, and bone pâté, as well as hydroxyapatite and BAG S53P4, the use of HA was discontinued after 18 cases, due to rejection and retraction occurring in 33% of the patients. Bone pâté (*N* = 33) showed pathological finding in 21%, the Palva flaps (*N* = 145) in 21%, primarily in the form of shrinkage (7%) and retractions (10%). The cartilage/bone pieces (*N* = 516) and BAG S53P4 (*N* = 133) only showed abnormal postoperative findings in 8% and 3%, respectively, concluding that a combination of BAG S53P4 and cartilage as cover is a suitable material for cavity obliteration.^[^ ^94^ ^]^

### 4.8 Oral and Maxillofacial

BAG S53P4 has shown to be a versatile bone substitute also in oral and maxillofacial surgery. It has been used in Le Fort I osteotomies (*N* = 25) in the treatment of bone gaps requiring bone grafting, in a retrospective observational case–control study evaluated data from medical records and CT scans of individuals who underwent Le Fort I osteotomy using a bioactive glass graft, when compared with nongrafted individuals. In this study BAG S53P4 did not optimally behave as a coadjuvant and favorable factor in bone formation and it could not be confirmed that the biomaterial played a fundamental role in bone healing, however it was innocuous to the maxillary sinus.^[^ ^95^ ^]^

In a prospective study BAG S53P4 granules were used as filling material in large mandibular advancement in bilateral sagittal split osteotomies (*N* = 25) in defects (8–15 mm) due to class II dentoskeletal deformities. The clinical and radiological results were good regarding to healing, bone regeneration, and stability of the osteotomy sites after a two‐years follow‐up. The recontouring of the inferior mandibular border provided a good soft tissue support and an excellent aesthetic outcome in 96%, showing that BAG S53P4 is a safe grafting material for osteotomy site defects providing long‐term stability at the osteotomy site and at the inferior mandibular border.^[^ ^96^ ^]^

In a prospective study on bone defects (*N* = 21, *N* patients = 20) in the maxilla or mandible filled with granules of BAG S53P4 for the treatment of benign tumors, cysts, or chronic infections to impacted teeth in the maxilla or mandible despite signs of chronic infection in the time of surgery. The outcome after a 2‐years follow‐up was successful in 20 patients despite an infection at the time of surgery in 65% of the patients. BAG S53P4 used in the treatment of large bone defects provides infection‐free reliable bone regeneration despite chronic infection at the time of surgery also improving the prognosis of adjacent teeth.^[^ ^10^ ^]^

In a prospective study on (*N* = 20) patients with an isolated blow‐out fracture of the orbital floor or with a combined zygomatico‐orbito‐maxillary complex fracture an anatomically drop‐shaped implant made of BAG S53P4 was used in the fracture treatment. The long‐term 32‐mont follow‐up of the implants showed successfully maintained orbital volume and compensated for the retrobulbar adipose tissue atrophy. None of the patients had any signs of complications related to the implant and the clinical outcome was very good.^[^ ^97^ ^]^ These results are in concordance with another study (*N* = 36) showing that the BAG S53P4 is a well‐tolerated material in orbital floor reconstruction.^[^ ^11^ ^]^

BAG S53P4 plates used in orbital floor reconstruction (*N* = 35), in orbital medial and superior walls reconstruction after fronto‐orbital trauma (*N* = 6), and after fronto‐orbital tumor resection (*N* = 8) have also shown to be a well‐tolerated and reliable reconstruction material alternative for reconstruction of orbital wall defects.^[^ ^98^ ^]^ These results are in concordance with favorable results (*N* = 28) showing that BAG S53P4 is well‐tolerated and promising repair material in the treatment of orbital floor fractures.^[^ ^99^ ^]^

Favorable results using BAG S53P4 (*N* = 17) together with autologous bone chips for sinus floor augmentation have also been reported thus decreasing the amount of bone needed.^[^ ^100^ ^]^ BAG S53P4 has also shown to be a good interposition graft in the repair of medium and large nasal septal perforations.^[^ ^101^, ^102^ ^]^

In the reconstruction of defects of the facial bones, BAG S53P4 granules and plates have been used in patients (*N* = 13) in 36 sites and compared to autograft bone grafts (*N* = 16) in the same patient. BAG S53P4 granules were used in facial bone defects in subperiosteal pockets and to obliterate frontal sinuses, whereas BAG S53P4 plates were used in orbital wall reconstruction. Clinical examination, middle face radiographs, and computed tomograms (CT) showed that the material was well tolerated.^[^ ^103^ ^]^

## 5 Conclusion

BAG S53P4 has emerged as a safe, effective, and durable bone graft substitute for the treatment of benign bone tumors, including enchondromas, aneurysmal bone cysts, and non‐ossifying fibromas in both adults and pediatric patients, demonstrating consistent results across a wide range of bone defect sizes and anatomical locations.

In spinal surgery, BAG S53P4 has shown its potential as a bone graft substitute both as a stand‐alone and when used with AB. Clinical studies across various spinal conditions and surgical techniques have reported achieved solid fusion rates. BAG S53P4 has also shown effectiveness and safety in pediatric scoliosis surgeries and in treating infected spinal conditions, achieving full fusion and recovery in infection‐related cases.

BAG S53P4 has also demonstrated good outcomes in trauma surgery, including depressed tibial plateau fractures, and non‐unions, with partial or full consolidation in most cases.

BAG S53P4 has proven to be a reliable, safe, and well‐tolerated material for frontal sinus obliteration in both short‐ and long‐term follow‐ups. Clinical studies on patients with chronic frontal sinusitis unresponsive to other treatments have shown successful sinus obliteration in most cases experiencing uneventful recovery. These findings support the long‐term safety and effectiveness of BAG S53P4 in frontal sinus surgery.

BAG S53P4 has shown strong potential in treating osteomyelitis and other complex bone infections as well as in diabetic patients. Clinical case reports and retrospective studies have demonstrated successful outcomes in managing osteomyelitis and diabetic foot infections with high bone healing rates, good integration of the material with bone, and resolution of infection. The results support its effectiveness and safety as part of limb‐salvage strategies in bone infection.

Equally good outcomes have been observed in long‐term studies in mastoid surgery demonstrating high rates of cavity obliteration, low complication and recurrence rates, and good anatomical preservation.

BAG S53P4 has demonstrated good healing results also in oral and maxillofacial surgery. Overall, BAG S53P4 appears to be a safe, effective, and flexible biomaterial for a range of reconstructive applications in the craniofacial region. Studies report that BAG S53P4 is generally well‐tolerated, supports bone regeneration, and shows good long‐term outcomes in aesthetics and functional stability.

In conclusion, the evidence strongly supports BAG S53P4 as a valuable biomaterial in orthopedic, trauma, spinal, ENT, and maxillofacial surgery. Its ability to promote bone growth, manage infection, and serve as a reliable void filler offers significant benefits in a multitude of clinical scenarios, often matching or exceeding traditional grafting materials while obviating donor site morbidity. While most studies indicate favorable outcomes, ongoing research, including randomized controlled trials, will further refine its applications and compare its noninferiority to other substitutes in specific contexts like large nonunion defects. Overall, BAG S53P4 stands out as a safe, efficacious, and versatile bone substitute with a broad and expanding role in reconstructive surgery.

Based on the excellent clinical results of BAG S53P4, new formulations are in development to extend the clinical indications. Nanoparticulate powder with higher biological and antimicrobial potency might be indicated for implant protection by prevention of bacterial adherence or biofilm formation or their eradication and initial results so far look promising. Additionally, new carriers for S53P4 BAG nanoparticulate powder resulting in the availability for creams and gel formulations that can potentially be utilized in soft tissue infections. These formulations would bind well to the soft tissue and enable a potent antimicrobial and angiogenic stimulus. Additionally, such formulations can also be applied as a bactericidal coating on the surface of orthopedic implants to prevent bacterial adherence.^[^ ^104^, ^105^, ^106^ ^]^

## Conflict of Interest

Lindfors NC, and Arts JJC are members of the advisory board of Bonalive.
